# Evidence of Highly Regulated Genes (in-Hubs) in Gene Networks of *Saccharomyces Cerevisiae*

**DOI:** 10.4137/bbi.s853

**Published:** 2008-07-14

**Authors:** Jesper Lundström, Johan Björkegren, Jesper Tegnér

**Affiliations:** 1 The Computational Medicine group, Center for Molecular Medicine, Department of Medicine, Karolinska Institutet, Karolinska University Hospital, Solna, SE-171 76 Stockholm, Sweden; 2 Division of Computational Biology, Department of Physics, Linköpings Institute of Technology, Linköping University, SE-581 83 Linköping, Sweden; 3 Clinical Gene Networks, Fogdevreten 2b, SE-17177, Stockholm, Sweden

**Keywords:** gene expression, network, algorithm, transcription regulation, protein-protein interactions, in-hubs

## Abstract

Uncovering interactions between genes, gene networks, is important to increase our understanding of intrinsic cellular processes and responses to external stimuli such as drugs. Gene networks can be computationally inferred from repeated measurements of gene expression, using algorithms, which assume that each gene is controlled by only a small number of other proteins. Here, by extending the transcription network with cofactors (defined from protein-protein binding data) as active regulators, we identified the effective gene network, providing evidence of in-hubs in the gene regulatory networks of yeast. Then, using the notion that in-hub genes will be differentially expressed over several experimental conditions, we designed an algorithm, the HubDetector, enabling identification of in-hubs directly from gene expression data. Applying the HubDetector to 488 genome-wide expression profiles from two independent datasets, we identified putative in-hubs overlapping significantly with in-hubs in the effective gene network.

## Introduction

In recent years, several shared features of biological networks, such as metabolic, protein–protein, and genetic networks, have been discovered (Barabási and Oltvai, 2004). Although most nodes (metabolites, proteins or genes) in these networks have few connections, some—referred to as “hubs”—have large numbers of connections. The discovery of hubs has led to superior immunization strategies (Pastor-Satorras and Vespignani, 2002) and improved efficiency in identifying protein interaction networks (Lappe and Holm, 2004). In addition the biological importance of protein hubs have been demonstrated by Jeong et al. (Jeong et al. 2001), where a correlation between high connectivity and lethality in yeast was found. Moreover, analysis of gene expression data and TF–DNA binding data has revealed that some TFs serve as out-hubs, thus regulating several other genes (Lee et al. 2002; Luscombe et al. 2004; Bergmann et al. 2004).

In contrast, the existence of in-hubs, genes having several incoming regulatory connections, has not yet been determined because of possible structural constraints limiting the number of TF binding sites in gene promoter regions. Analysis of TF–DNA binding data has shown a narrow in-degree distribution in the transcription regulation network (Lee et al. 2002).

Another limitation which also motivates our analysis is that since our current knowledge as represented by these data sources, is most likely incomplete, it would therefore be advantageous to find novel in-hubs directly from gene expression data. However, it has not yet been possible to identify in-hubs directly from gene expression data by using network reconstruction algorithms because these algorithms suffer from a complexity problem (Gardner and Faith, 2005; Tegnér et al. 2003; Tegnér and Björkegren, 2007; Bansal et al. 2007; Han et al. 2004). As an illustration, consider the problem of identifying all incoming connections 3 to a given gene. This corresponds to choosing k predictive genes out of *N* possible, which can be done in $\left(\begin{array}{l} N\\ k \end{array}\right)$ ways. For example, there are ~10^13^ different possibilities for how 10 incoming connections can be wired into a single gene in a 100 gene network. To come to terms with this complexity problem, network reconstruction algorithms as a rule limit the maximal in-degree (*k_max_*) for each gene to a small number (i.e. *k_max_* < 5 or less). Thus, current network identification algorithms do not have the power to detect in-hubs from gene expression data. It is therefore desirable to develop algorithms that can directly detect in-hubs from gene expression data and thereby increase the power of current network identification algorithms.

The paper is organized as follows. In the first part of we assess the existence of in-hubs in the yeast network by integrating transcription and protein–protein binding data into what we refer to as the effective gene network. Then, in the second part we develop an algorithm—the HubDetector—which can identify in-hubs as defined by the effective gene network directly from gene expression data. The HubDetector is evaluated *in silico* and validated using two different gene expression datasets from yeast.

## Results

### Identification of an effective gene to gene network by data integration

**Figure 1:**
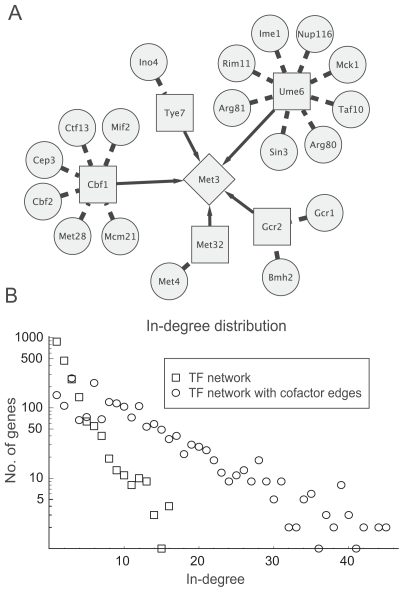
Proteins that bind to TFs influence gene expression. (**A**) A schematic illustration of regulation of Met3, a gene involved in sulphate assimilation. Shown are its five TFs (Met32, Cbf1, and Gcr2, Ume6 and Tye7) (squares) and proteins binding to these TFs (circles) (MacIsaac et al. 2006; Deane et al. 2002; Xenarios et al. 2000). Considering proteins binding to TFs as transcription co-factors result in 23 proteins regulating MET3 expression. (**B**) The number of incoming regulatory edges for S. cerevisiae genes. and the number of incoming regulatory edges, considering the TF network alone (squares) and with proteins binding to TF influencing TF activity (circles).

In a gene network the genes are nodes and directed edges represent regulatory interactions between regulators and a target genes. This is in contrast to edges in the transcription factor network since the edges in a gene regulatory network often involve multiple complex interactions involving indirect pathways of protein and metabolites (Brazhnik et al. 2002; Blais and Dynlacht, 2005). One example of protein–protein interactions mediating signals from a regulator via a TF to a target gene is found in the galactose utilization pathway, in which the TF Gal4p is modulated by Gal3p and Gal80p (Blais and Dynlacht, 2005; Ideker et al. 2001). This principle is illustrated by analysis of the MET3 network (Fig. 1a). When only the strict TF network was considered, no gene had more than 16 incoming edges (Fig. 1b). However, when proteins binding to a TF were considered as active cofactors some genes had 40 or more incoming edges. Importantly, most genes had few edges regardless of whether the strict TF network or protein–TF interactions were considered. To further justify that it makes biological sense to include proteins binding to TFs we assessed the fraction of genes annotated to the gene ontology category ”transcription regulation activity”. Table 1 shows cofactor proteins are known to be involved in transcription regulation in 26.7% of the cases. This is less than the well characterized set of TFs (68.7%) but significantly higher than for all genes (7.1%; *P* < 10^−10^).

**Figure 2:**
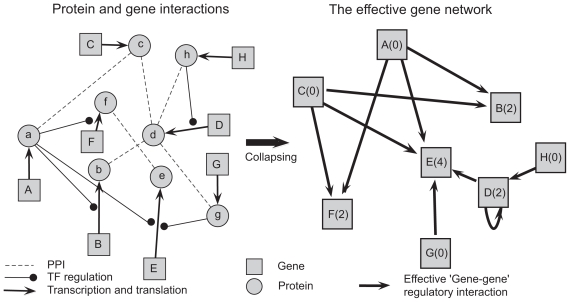
Collapsing protein–protein and TF–DNA networks into an effective gene network. When the network of interactions between eight hypothetical genes and their respective protein neighbors (left) is collapsed into an effective gene network (right), each protein interaction with a TF constitutes a regulatory edge to the TF targets. For example, in the gene–protein network, protein d interacts with TF g, which in turn regulates the expression of gene E. In the effective gene network, gene D is therefore a regulator of the expression of gene E. The number of regulating connections (in-degrees), is shown in parenthesis within the gene symbols in the effective gene network.

To calculate the number of incoming edges to a given gene in the yeast network, we collapsed the protein–protein and transcription networks into what we refer to as the effective gene network (Fig. 2 and Methods). By defining the effective gene network in yeast from these data sources, we could calculate the average number of connections between all yeast verified ORFs (Cherry et al. 1998), the 300 most-regulated genes (in-hubs), and 237 most regulating genes (out-hubs) (Table 1). In-hubs had an average of 24.4 incoming edges, whereas out-hubs had only slightly more incoming edges than the average for all genes (6.0 versus 4.2). Moreover, 46.4% of the out-hubs, but only 12.6% of the in-hubs, were involved in transcription regulation. Thus, by integrating currently available data of TF and protein binding, we find evidence for the existence of in-hubs in the regulatory network of yeast. Moreover, out-hubs (many of which are TFs) appeared to be mostly distinct from in-hubs.

### Design and evaluation of the HubDetector algorithm in silico

**Figure 3:**
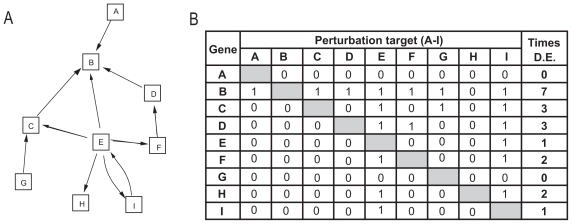
Design of the HubDetector algorithm. HubDetector is based on the rather simple idea that genes that are differentially expressed often in different internal/external conditions are more likely to be in-hubs. The rationale for this hypothesis is illustrated with a simple network (**A**) consisting of nine nodes with one out-hub (gene E) that regulates genes B, C, F, H, and I and one in-hub (gene B) that is regulated by genes A, C, D, and E. In this network, the HubDetector should detect gene B if gene expression measurements are obtained after other genes in the network are perturbed. Arrows indicate regulatory interactions. The table (**B**) shows which gene will be differentially expressed (D.E.) given different perturbation targets in the network. Gene B (the in-hub) is differentially expressed most frequently across all targets.

In-hubs have been difficult to detect directly from gene expression data. There are several explanations for this. Current approaches for identifying networks from gene expression datasets cannot detect in-hubs, since the combinatorial complexity is too great when there are several in-coming edges (Gardner and Faith, 2005; Tegnér et al. 2003; Tegnér and Björkegren, 2007; Bansal et al. 2007; Han et al. 2004). Moreover, our current knowledge of the yeast network is based on available experimental datasets and is therefore incomplete. Moreover, different cellular conditions activate different subsets of in-hubs, leaving some inactive and undetectable (Luscombe et al. 2004). To identify active in-hubs under different cellular conditions directly from gene expression data, without prior knowledge of the architecture of the gene network, we designed an algorithm referred to as HubDetector. The HubDetector algorithm is based on the idea that perturbations, (genetic or changes to the cellular environment) more often affect the transcription of in-hubs than other genes in the network, due to the larger number of incoming edges (Fig. 3).

**Figure 4:**
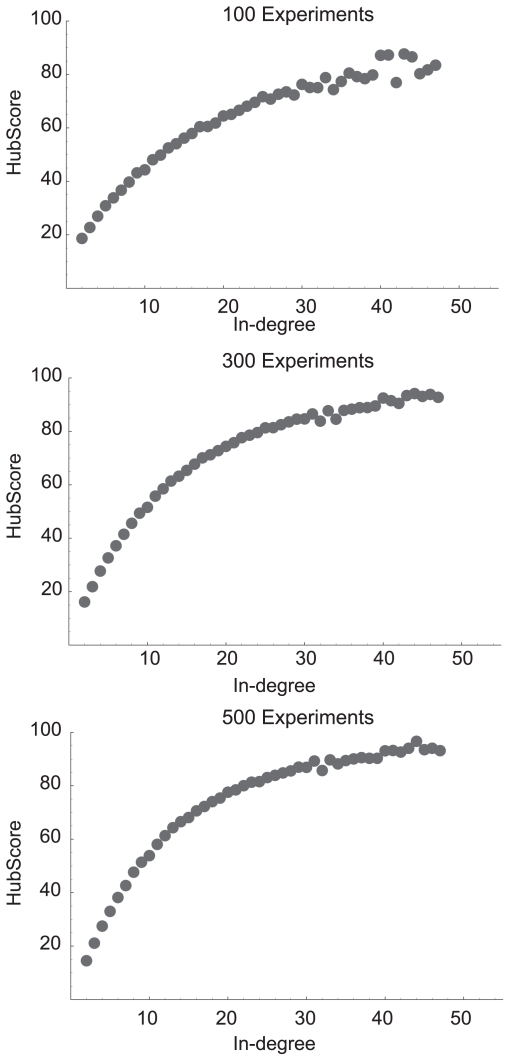
The HubScore as a function of in-degree in simulated gene networks after 100, 300 and 500 single gene perturbation experiments. The results are based on simulations of gene networks with a gaussian in-degree distribution and *σ_noise_* = 0.002.

Using random *in-silico* networks to generate simulated gene expression data from a well defined network structure allowed systematic evaluation of the algorithm. The HubDetector was tested on *in-silico* networks, each containing 4000 nodes (similar to the number of genes in yeast) (Fig. 4). The HubDetector performance converges after around 300 experiments depending on network structure and noise (Fig. 4 and Table 2). Moreover, Table 2 shows simulation results when changing network architecture, noise level and perturbation strategy. The HubDetector is robust against alterations in network architecture and noise level. We observe that the performance of the HubDetector is better for lower noise levels. A perturbation strategy which randomly perturbs multiple targets (called “environmental” in Table 2) appear to be beneficial to both performance and convergence rate. Thus the HubDetector could be applied to datasets with single gene perturbations or environmental states.

### Validation of the HubDetector using gene expression data

**Figure 5:**
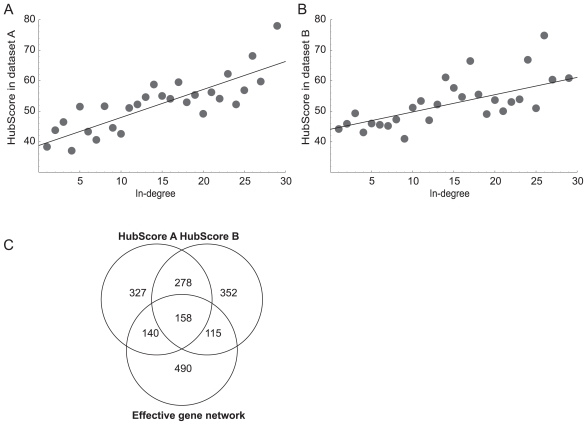
Validation of HubDetector using two independent gene expression datasets from S. cerevisiae. (a–b) The number of regulatory interactions as calculated from the effective gene networks, shown as a function of the HubScore generated by the HubDetector. HubDetector was applied to two datasets: (**A**) 273 gene expression profiles of 300 generated from mostly nonlethal gene deletions (Hughes et al. 2000) and (**B**) 215 expression profiles generated from yeast cultures with titratable promoters of genes essential for cell survival (Mnaimneh et al. 2004). The effective gene network in-hubs were identified as described in Figure 2 and Methods. (**C**) Venn diagram illustrating the intersections of gene-sets predicted to be in-hubs and the gene-set with most regulators in the effective gene network. The two circles above represent the genes with highest HubScore (top 20%) in dataset A and B respectively. The circle below represent the genes with most in-edges (top 20%) in the effective gene network.

To further validate the HubDetector we analyzed to two publicly available whole-genome yeast expression datasets. From dataset A (Hughes et al. 2000), we used 273 of 300 gene expression profiles generated from mostly nonlethal deletions. Dataset B (Mnaimneh et al. 2004), generated more recently, consisted of 215 expression profiles from yeast cultures with titratable promoters of genes essential for cell survival. From each dataset, HubDetector generated a list of genes ranked according to their HubScores (see Supplementary Data on-line). The observed relation between the HubScore and the number of regulatory interactions of each gene in the effective network (Fig. 5a and b) clearly demonstrates that the HubDetector identifies in-hubs. The correlations between the HubScore and the number of regulatory interactions are 0.18 (*P* < 10^−32^) and 0.11 (*P* < 10^−13^) for expression dataset A and B respectively. The observed correlations were found to be robust against errors in the TF binding data. The two gene expression datasets (A and B) are based on perturbations of essential versus non-essential genes. The propagation of gene activity throughout the networks should therefore differ (Luscombe et al. 2004). The HubDetector was expected to predict two subsets of in-hubs from each dataset. Surprisingly the genes that had a high HubScore in dataset A or B (upper 20th percentile or 903 genes in each dataset) overlapped by a 48.3% (Fig. 5c).

## Discussion

In this study we considered proteins binding to transcription factors acting as transcription co-factors and thus regulating the target gene of that TF. The function of proteins binding to TFs acting as functional cofactors were supported by Gene ontology analysis revealing an over-representation of gene ontology function “Transcription regulator activity”. Our analysis uncovered a significantly broader in-degree distribution of the target genes, thus supporting the existence of in-hubs in gene regulatory networks of yeast.

To enable the prediction of in-hubs directly from gene expression data we introduced an algorithm, the HubDetector. Validation of the HubDetector using two distinct gene expression datasets independently showed statistically significant correlations between the HubScore and the number of incoming edges in the effective gene network. It should be noted that the genes that the HubDetector identifies are the genes changing expression most often between different cellular states these, differ from the genes with the highest variance as studied in Bilu and Barkai (2005).

Interestingly, several of the identified in-hubs from the expression data were identical to in-hubs recovered from the integration of protein–protein interactions with TF–DNA binding data. It should be noted the HubDetector also identifies several putative and novel in-hubs which do not have many incoming edges in the effective network which was constructed in the first part of this work.

Rung et al. (2002) proposed a disruption network reconstruction algorithm based on the idea that whenever the expression level of a gene is changed by the deletion of another gene a putative interaction has been identified. Our study reveals that it is possible to interpret some hubs in a disruption network as putative in-hubs. However, in contrast to the disruption network reconstruction, the HubDetector is applicable not only to specific perturbations like gene deletions but also to non-specific perturbations like distinct cellular or environmental states.

Using the HubDetector does not require a complete identification of all interactions within the network. Thus, the HubDetector bypasses the limitations of current high-resolution network identification algorithms since the $\left(\begin{array}{l} N\\ k \end{array}\right)$ problem is avoided. It should also be noted that current modular analysis techniques (e.g. Segal et al. (2005)) do not detect in-hubs. An interesting future application of the HubDetector is to use it prior to network reconstruction in order to obtain an estimate of the in-degree distribution in a biological network and predict genes for which a higher *k* – *value* (in-degree) is required in the network reconstruction algorithm. Thus, the stage is set to systematically map in-hubs by applying HubDetector to the increasing number of publicly available whole-genome expression datasets.

Out-hubs (many of which are TFs) are important regulators of cellular activities and therefore are considered to be good drug targets (Butt and Karathanasis, 1995). In-hubs are instead highly regulated, maybe acting as sensors of intra- and extra-cellular environments, such as altered growth conditions or unfavorably states (i.e. diseases), and providing this information to the appropriate out-hubs. Thus, in-hubs merit further attention in future studies.

## Materials and Methods

### Gene identifiers

The analysis in this study is restricted to verified ORFs, symbols and names for 4517 ORFs where downloaded from from Saccharomyces Genome Database (Cherry et al. 1998) in February 2007.

### Construction of the effective gene network in yeast

Transcription regulation interaction data were downloaded from http://fraenkel.mit.edu/improved map/orfs by factor.tar.gz in January 2007 (MacIsaac et al. 2006). In our analysis we are using the 4900 edges between 2022 genes described in the file orfs by factor p 0.005 cons2.txt. Protein–protein binding data were collected from the Database of Interacting Proteins (DIP) in March 2007 (Xenarios et al. 2000), only including the high-confidence interactions (Deane et al. 2002) resulted in a Protein–Protein interaction (PPI) network involving 5562 interactions between 2310 proteins.

We used the edges in the transcription network to define a first set edges in the effective gene network. Then, for a given TF node in the transcription network, we identified all the proteins that bind to that TF. For this TF, a second set of edges are added from each binding protein to each target gene which is regulated by the TF via a primary edge. By repeating this procedure for all TFs an effective gene network was constructed. The effective gene network, constructed from the transcription network and protein–protein datasets referred to above, has 18 949 regulatory edges and 2085 genes connected by at least one edge.

### In-silico gene network architecture and computational model

In agreement with previous experimental studies of biological networks, we used a wide out-degree distribution of the *in-silico* networks (Barabási and Oltvai, 2004). Two types in-degree distributions where analyzed, power law and gaussian. The construction of networks with power law degree distribution follows the procedure as described by (Wagner, 2002), using *τ* = 1.8, *γ* = 1000, and the maximal in-degree and out-degree was 400. Networks with a gaussian in-degree distribution are drawn from the non-negative part of *N*(−50,30). The network was constructed by randomly connecting nodes approximating the above in-degree and out-degree distribution. Genes are connected with weighted directed edges. The weights representing regulatory strengths are sampled from two normal distributions *N*[10,3] and *N*[−10,3] with equal probability. The diagonal of the connectivity matrix is subtracted by a decay term representing degradation of mRNA. The degradation terms are 10% larger than the largest eigenvalue for the connectivity matrix, thereby ensuring stability of the final system. The gene regulatory dynamics follows a system of linear differential equations:

(1) $$\frac{dx}{dt}=Ax+p$$

where *x*(*t*) is expression vector at time *t* and *A* is the connectivity matrix with *a_i,j_* as the regulatory strength of gene *j* on gene *i* (*a_ij_* = 0 if *j* does not regulate *i*).

### Generation of in-silico gene expression data

Without any external force on the system (*P* = 0), we obtain the trivial solution (*x* = 0), which corresponds to the wild-type steady state. To design an algorithm for detecting hubs from experimental perturbation data, we simulate perturbations by changing the stimuli (*P*) on the right-hand side of the equation. The euclidean length of the perturbation is always 1.0. For “Single gene” perturbations only one element (*P_i_*) is changed while ”environmental” perturbations refer to a procedure where a random number of genes are perturbed simultaneously. (Drawn from the non negative part of the normal distribution *N*[5.0, 2.0] for each experimental profile.) After perturbation the new steady-state solution is found by solving the linear equations.

White noise is added to the expression values before differentially expressed genes are identified. The noise is normally distributed (around 0) with standard deviation *σ_noise_*. Thus the noise level can be varied using different values of *σ_noise_*. Knowledge of the noise level enables us to compute a cutoff which will result in a predetermined false positive rate (0.01). Which results in a specificity in the range 0.6–0.9 for cases presented in this article, however this depends on the actual number of affected genes and *σ_noise_*. All simulation results in this article are based on 20 repetitions with different instantiations of the gene network.

### Genome-wide expression datasets

Two public S. cerevisiae genome-wide expression datasets were used. Dataset A (Hughes et al. 2000) contains 300 genome-wide expression profiles mainly from gene deletions. Aneuploid deletion strains and repeated deletions were removed (Supplementary Table on-line), leaving 273 perturbation experiments. We used the error model from (Hughes et al. 2000) and a threshold p-value of 0.01 to find Differentially expressed genes. Dataset B (Mnaimneh et al. 2004) consists of genome-wide expression profiles from perturbations of 215 expression profiles generated from yeast cultures with titratable promoters of genes essential for cell survival. Since dataset B did not include control experiments to estimate gene variance we instead regarded genes with twofold expression change (perturbed state versus wild-type) as differentially expressed.

### HubDetector algorithm

The HubDetector algorithm ranks the genes according to the number of times they are differentially expressed across a set of expression profiles. The HubScore for gene *g* is computed as:

*DE_g_* ← 0 *and HubScore_g_* ← 0 *for g* ∈ *G*

*for each g* ∈ *G*

*for each p* ∈ *P*

if g is differentially expressed in p*

*DE_g_* + +

*for each g*_1_ ∈ *G*

*for each g*_2_ ∈ *G*

*if DE_g_*_1_ > *DE_g_*_2_

*HubScore_g_*_1_ + +

*HubScore_g_*_1_—*HubScore_g_*_1_/*N*

Where *G* is the set containing *N* measured genes and *P* is the set of all expression profiles. The line marked (*) is a function determining whether gene *g* is differentially expressed in profile *p* (i.e. a fold change threshold or a statistical test).

### Statistics

P-values based on spearman rank correlation is computed through a Fisher transformation. P-values for enriched gene sets (GO-analysis) are estimated using hypergeometric distributions.

### Software

Computations and simulations were performed in Mathematica 5.2 (Wolfram Research, 2005).

## Supplementry Materials

### Additional Files

#### RemovedProfiles.pdf—supp. Table: Expression profiles not used in analysis

A list of twenty seven expression profiles where removed before analyzing the expression dataset by (Hughes et al. 2000).

**Table S1:** Twenty-seven expression profiles removed from the original expression dataset of Hughes et al. (2000). For detailed description of all 300 expression profiles see on-line material for Hughes et al. 2000.

Experiment #	Deleted genes(s)	Reason for removing
67	fus3 and kss1	There are experiments where these genes are deleted separately present in the dataset.
89	isw1 and isw2	
41,42	dig1 and dig2	
243	yml033w/yml034w	Two experiments perturb the same gene. Only one experiment is kept.
62, 127	fks1 ras1	
14,16,18,20,21,46,48,56,87,118,131,133, 137,138, 141,143,171,230, 272, 275	–	Aneuploid mutant strains.

#### HubScore.tsv—supp.data HubScore

HubScores.tsv reports the HubScore for all genes. The three columns are 1) systematic gene names 2) HubScore in Hughes et al. (Hughes et al. 2000) 3) HubScore in Mnaimneh et al. (Mnaimneh et al. 2004).



## Figures and Tables

**Table 1:** Hub-genes in the effective regulatory network.

Groups	No. genes	In-degree	Out-degree	Transcription regulator activity GO:0030528
All	4517	4.2	4.2	7.1%
In-hubs	300	24.4	5.4	12.6%
TFs	114	7.4	81.4	68.7%
Cofactors	123	4.7	92.7	26.7%
Out-hubs (TFs cofactors)	237	6.0	87.3	46.4%

**Table 2:** HubDetector performance on gene expression profiles generated from linear network models with 4000 nodes.

In-degree distribution	Noise (*σ_noise_*)	Perturbation strategy	Spearman rank correlation		
			100 profiles	300 profiles	500 profiles
Power-law	0.001	single gene	0.54 ± 0.06	0.58 ± 0.04	0.59 ± 0.04
Power-law	0.002	single gene	0.46 ± 0.09	0.51 ± 0.07	0.54 ± 0.05
Power-law	0.005	single gene	0.34 ± 0.08	0.40 ± 0.07	0.43 ± 0.07
Gaussian	0.001	single gene	0.71 ± 0.06	0.80 ± 0.03	0.82 ± 0.03
Gaussian	0.002	single gene	0.52 ± 0.09	0.68 ± 0.07	0.73 ± 0.06
Gaussian	0.005	single gene	0.19 ± 0.08	0.31 ± 0.08	0.38 ± 0.10
Gaussian	0.001	environmental	0.78 ± 0.03	0.83 ± 0.02	0.84 ± 0.02
Gaussian	0.002	environmental	0.57 ± 0.12	0.69 ± 0.07	0.74 ± 0.06
Gaussian	0.005	environmental	0.21 ± 0.09	0.32 ± 0.11	0.38 ± 0.11
